# An Evaluation of the Impact of ^60^Co Irradiation on Volatile Organic Compounds of Olibanum Using Gas Chromatography Ion Mobility Spectrometry

**DOI:** 10.3390/molecules29071671

**Published:** 2024-04-08

**Authors:** Qiao Luo, Shanshuo Liu, Ye He, Jiayao Liu, Xinyu Zhang, Liqiu Zheng, Dan Huang

**Affiliations:** 1 The First Hospital of Hunan University of Chinese Medicine, Hunan University of Chinese Medicine, Changsha 410007, China; kakuky0727@163.com; 2 State Key Laboratory of Chinese Medicine Powder and Medicine Innovation in Hunan (Incubation), Science and Technology Innovation Center, Hunan University of Chinese Medicine, Changsha 410208, China; hzdlss2002@163.com (S.L.); hy070915@126.com (Y.H.); liujiayao4777@163.com (J.L.); zhangxinyu202401@163.com (X.Z.); zhengliqiu202401@163.com (L.Z.)

**Keywords:** olibanum, ^60^Co irradiation, volatile organic compounds, gas chromatography ion mobility spectrometry

## Abstract

Olibanum is a resinous traditional Chinese medicine that is directly used as a powder. It is widely used in China and is often combined with other traditional Chinese medicine powders to promote blood circulation and relieve pain, as well as to treat rheumatism, rheumatoid arthritis, and osteoarthritis. Powdered traditional Chinese medicine is often easily contaminated by microorganisms and ^60^Co irradiation is one of the good sterilization methods. Volatile organic compounds (VOCs) are the main active ingredient of olibanum. The aim of this study was to validate the optimum doses of ^60^Co irradiation and its effect on VOCs. ^60^Co irradiation was applied in different doses of 0 kGy, 1.5 kGy, 3.0 kGy, and 6.0 kGy. Changes in VOCs were detected using gas chromatography ion mobility spectrometry. A total of 81 VOCs were identified. The odor fingerprint results showed that, with an increase in irradiation dose, most of the VOCs of olibanum changed. Through principal component analysis, cluster analysis, and partial least squares discriminant analysis, it was demonstrated that, at 1.5 kGy, the impact of radiation on the VOCs of olibanum was minimal, indicating this is a relatively good irradiation dose. This study provides a theoretical basis for the irradiation processing and quality control of resinous medicinal materials such as olibanum and it also provides a good reference for irradiation technology development and its application to functional foods, thus making it both significant from a research perspective and useful from an application perspective.

## 1. Introduction

Traditional Chinese medicinal powders are made from crushed materials. Medicinal powder is an important ingredient in many traditional Chinese medicines and it is also widely used in health foods, such as Panacis Quinquefolii Radix powder, Notoginseng Radix ET Rhizoma powder, and Gastrodiae Rhizoma powder.

Traditional Chinese medicine powders are important dosage forms of traditional Chinese medicine. In addition to the more common granules, certain Chinese medicine tablets and capsules are made directly from Chinese medicine powder. The 2020 edition of the Chinese Pharmacopoeia recorded 666 types of powder preparations, which accounted for 57.07% of all traditional Chinese medicine preparation products.

Due to the high bacterial content of traditional Chinese medicine powder, it must be sterilized before use in the production of preparation products [1]. Therefore, the sterilization process is an important part of producing traditional Chinese medicine powder preparations. Currently, increasing attention is being paid to the impact of the sterilization process on a product’s quality.

Commonly used traditional Chinese medicine sterilization methods include heat sterilization (such as dry heat sterilization, moist heat sterilization, saturated steam sterilization, and superheated steam sterilization) and non-heat sterilization (drug sterilization, such as ethylene oxide sterilization, hydrogen oxide sterilization, etc., as well as ultraviolet sterilization and radiation sterilization) [2]. The characteristics of commonly used traditional Chinese medicine sterilization methods are shown in Table 1.

Olibanum is the bark of *Boswellia carterii* Birdw. or *Boswellia bhaw-dajiana* Birdw. There are approximately 30 species of it in the world and they are mainly cultivated in Ethiopia and Somalia [3]. Olibanum has a special aroma. Furthermore, it is used to promote blood circulation, emote blood stasis, disperse swelling, and promote tissue regeneration. Moreover, it is often used for chest disorders, heart pain, pain in the stomach duct, dysmenorrhea, amenorrhea, postpartum stasis and obstruction, abdominal pain caused by masses, rheumatalgia (i.e., the localized or systemic pain caused by diseases of the joints, muscles, bones, and tissues around the joints), hypertonicity of the sinews and vessels, traumatic injuries, swelling abscess, sores, and ulcers [4]. Clinically, it is also used for antibacterial [5,6,7], anti-tumor, and antiviral effects [8], as well as for the treatment of rheumatism, rheumatoid arthritis, and osteoarthritis [9].

As resinous medicinal materials soften when exposed to heat and become more viscous, heat sterilization is not suitable. Certain non-heat sterilization methods may produce unwanted drug residues or incomplete sterilization. This makes irradiation sterilization one of the most reliable sterilization methods for resinous medicinal materials. Irradiation sterilization is a cold sterilization method that uses highly penetrating gamma rays produced by ^60^Co, the main mechanism of which is destroying the DNA and RNA in microbial cells. This causes the damaged DNA and RNA to degrade, which causes the organisms to lose their ability to synthesize proteins and maintain their genetic functions, thereby exerting a bactericidal effect on microorganisms [10].

The maximum acceptable dose should be determined as the highest dose that does not affect the safety, efficacy, or stability of the drug during sterilization. The main parameter of radiation sterilization is the radiation dose and it should be as low as possible while still having a bactericidal effect. When performing radiation sterilization, it is necessary to determine the maximum and minimum dose values for the sterilization process, which involves evaluating the relationship between its dose values and the maximum and minimum dose values via means of dose distribution tests. Biological monitoring and periodic dose audits are also performed during sterilization to ensure the effectiveness of radiation sterilization and the continued validity of the dose. 

The radiation dose absorbed by the sterilized item is monitored by the use of dosimeters, the placement of which is determined empirically to adequately verify that the dose absorbed by the sterilized item is within the specified limits. Dosimetry complies with national and international standards. 

Irradiation has high sterilization efficiency and does not cause the temperature of the irradiated object to rise too significantly. It is particularly suitable for heat-sensitive and volatile products. Radiation sterilization has been widely used in foods and Chinese medicinal materials [11,12,13]. 

The chemical composition of some medicinal materials will change to a certain extent after being sterilized via irradiation at different doses. However, there are still only a few systematic studies on olibanum exposed to different irradiation doses. Gas chromatography ion mobility spectrometry (GC-IMS) is a highly adaptable rapid analysis technology with high sensitivity and high separation capability and it is widely used in the food industry, clinical medicine, traditional Chinese medicine, and other fields [14,15,16,17,18]. Olibanum contains a large number of volatile organic compounds (VOCs). GC-IMS can separate and identify various VOCs in olibanum samples. Understanding the types and contents of these compounds is crucial for evaluating the quality and authenticity of olibanum. Testing irradiated olibanum samples is more helpful in understanding the impact of irradiation treatment on VOCs in olibanum.

In 1997, the Chinese Ministry of Health issued the “^60^Co Irradiated Traditional Chinese Medicine Sterilization Dose Standard” (as an internal trial). This standard stipulates that the irradiation dose of traditional Chinese medicine material powder shall not exceed 6.0 kGy, while the irradiation dose of olibanum shall not exceed 3.0 kGy. The State Food and Drug Administration issued the “Technical Guidelines for Irradiation Sterilization of Traditional Chinese Medicines” in 2017, which recommends that the maximum overall average irradiation dose, in principle, of traditional Chinese medicines should not exceed 10.0 kGy in principle. The recommended irradiation dose for the powder of semi-finished traditional Chinese medicine containing olibanum should not exceed 3.0 kGy. 

However, there has been no comprehensive evaluation of the changes in the components of olibanum caused by high-intensity irradiation. VOCs are the main active components of olibanum. Therefore, this study used GC-IMS combined with chemometrics to analyze the VOCs of olibanum, thereby aiming to explore the effects of ^60^Co irradiation at different doses (i.e., 0 kGy, 1.5 kGy, 3.0 kGy, and 6.0 kGy, which are represented by RX-01, RX-02, RX-03, and RX-04, respectively). The influence of irradiation on VOCs in olibanum provides a theoretical basis for the irradiation processing and quality control of olibanum and other resinous medicinal materials and it also provides new research ideas and methods for the modern processing and quality control of traditional Chinese medicine.

## 2. Results

### 2.1. GC-IMS Profiles of Olibanum at Different Irradiation Doses

**Figure 1:**
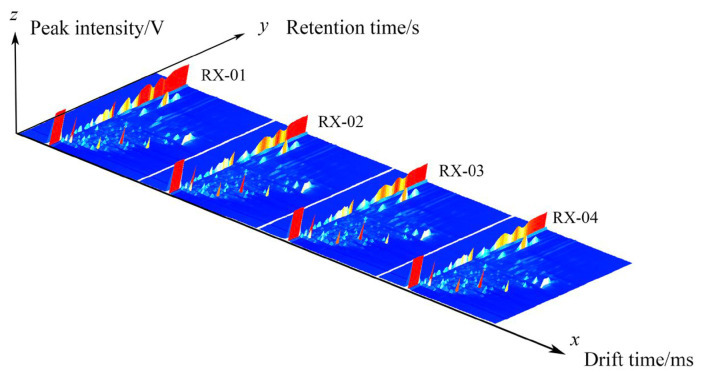
The three-dimensional spectra of the VOCs of the four groups of olibanum.

The three-dimensional spectra of VOCs at ^60^Co doses of 0 kGy, 1.5 kGy, 3.0 kGy, and 6.0 kGy are shown in Figure 1 and they are represented by RX-01, RX-02, RX-03, and RX-04, respectively. The *x*, *y*, and *z* axes in the figure represent the migration time, gas chromatography retention time, and peak intensity, respectively. It can be seen from the three-dimensional spectra of the four groups of olibanum that there are certain differences in the olibanum peak signal intensity under different irradiation doses, thus indicating that there are certain differences in the content of olibanum volatile oil in each group. The height of the red bulge represents the intensity of the signal of the component in olibanum. The part with a high red bulge, which corresponds to a high content of the component, indicates that the signal was strong. The part with a low red bulge, which corresponds to a low content of the component, indicates that the signal was weak. 

**Figure 2:**
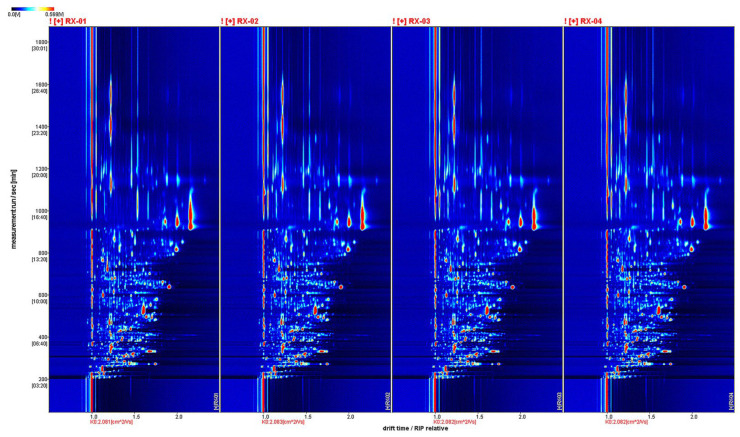
The two-dimensional spectra of the VOCs of the four groups in four groups of olibanum.

By projecting the three-dimensional GC-IMS spectra, four groups of two-dimensional GC-IMS images of olibanum can be obtained, as shown in Figure 2.

VOCs are represented by dots on either side of the RIP. The color indicates the concentration of the substance, with darker colors indicating higher concentrations. The background of the figure is blue and the red vertical line at 1.0 is the reactive ion peak (RIP). The retention time(s) of the gas chromatogram corresponds to the ordinate and the ion migration time (normalized processing) corresponds to the abscissa.

The background of the figure is blue and the red vertical line at 1.0 is the RIP (reactive ion peak). 

We selected the spectrum of the RX-01 sample as a reference and subtracted the reference from the spectra of other samples to obtain a comparison chart of the differences between the samples. If the volatile organic matter content in the target sample and the reference were the same, then we subtracted the reference. The background was set as white and the presence of red meant that the concentration of the substance in the target sample was higher than the reference, whereas the presence of blue meant that the concentration of the substance in the target sample was lower than the reference. By comparing the two-dimensional spectra, the difference in the concentration of the volatile substances in each sample could be visually observed. 

**Figure 3:**
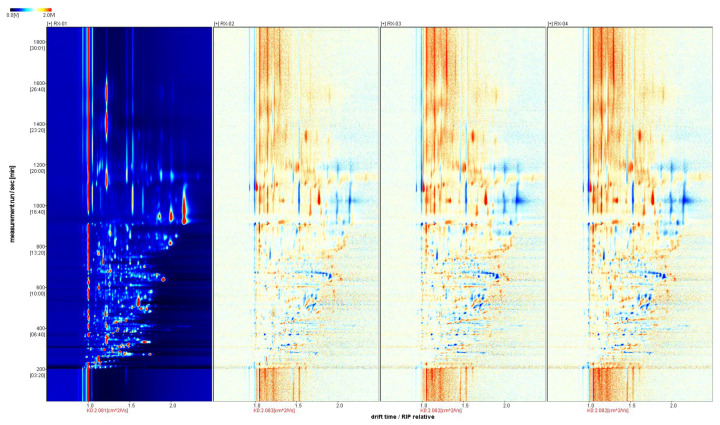
Analysis of the spectral differences between RX-01 and the other three groups of olibanum.

The three coordinate axes represent the migration time (*x*-axis), the retention time (*y*-axis), and the signal peak intensity (*z*-axis). From the figure, you can intuitively observe the differences in the volatile organic compounds in the different samples. From Figure 3, it can be seen that a blue color exists in the right-side region of RX-02, RX-03, and RX-04, which means that the concentration of the substance is lower than the reference concentration. In addition, a red color is found in the left side region, which means that the concentration of the substance is higher than the reference. It can be concluded that there are large differences in the content of the multiple VOCs, which is consistent with the results that were obtained from the 3D spectra and the top view.

### 2.2. Qualitative Analysis of the VOCs in Olibanum

**Figure 4:**
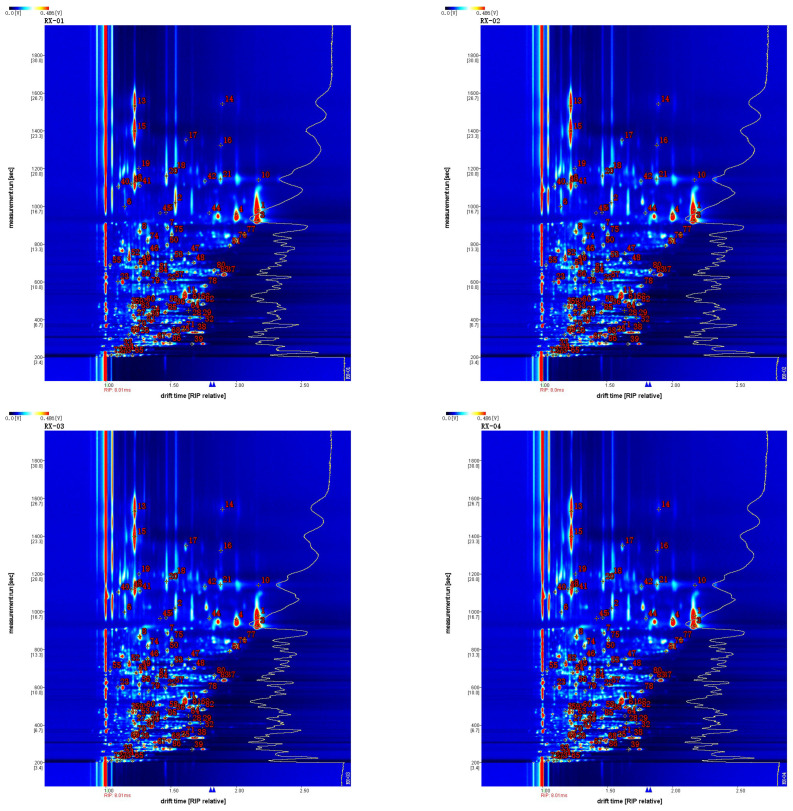
Characteristic peak position plot of the VOCs of olibanum with different irradiation doses. Note: RI is the retention index; Rt is the retention time; Dt is the migration time; and RIP rel refers to the normalization process.

In the GC-IMS two-dimensional spectrum, there were certain differences in the VOCs. Combined with the NIST and IMS databases that were built into the software, a qualitative analysis of VOCs was performed, the ion mobility spectrum of which is shown in Figure 4. Each point represents an organic substance and was qualitatively searched in the database based on its corresponding 2D data. The drift time is expressed on the abscissa and the retention time is expressed on the ordinate. A total of 81 VOCs were detected in this study, including 20 alcohols, 20 esters, 14 aldehydes, 13 ketones, 7 terpenes, 3 olefins, 2 pyridines, and 2 acids. The compounds 1-butanol, 3-methyl-, and acetate are represented by serial numbers 31 and 32 and 2-ethyl hexanol is represented by serial numbers 44 and 45 in Table 2, Table 3. All of these have the same retention time and it is now generally accepted that these compounds can be accurately characterized when the monomer and dimer are present at the same time.

### 2.3. Fingerprint Analysis of the Volatile Organic Compounds of Olibanum at Four Irradiation Doses

**Figure 5:**
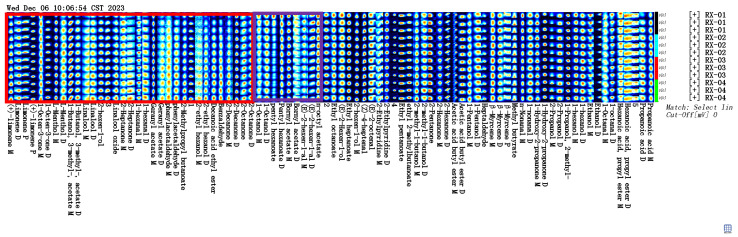
Gallery plot of the VOCs selected via GC-IMS.

The VOC fingerprints of the four groups of irradiation doses of olibanum are shown in Figure 5. The GC-IMS analysis showed that the olibanum VOCs’ possessed higher contents of esters, ketones, and alcohols, followed by aldehydes. The fingerprint pattern showed that, as the irradiation intensity increased, the contents of limonene, 1-octen-3-one, menthol, isoamyl acetate, linalool, 2-hexene, linalool oxide, 2-heptyl, ketone, hexanal, geranyl acetate, phenylacetaldehyde, isobutyl butyrate, 2-ethylhexanol, ethyl decanoate, benzaldehyde, 2-decanone, and 2-octanone all decreased. RX-04 was found to have the highest content. However, as the irradiation intensity increased, the contents of octanol, amyl hexanoate, bornyl acetate, and (E)-2-hexenal gradually decreased, with the highest contents being found in RX-01, as shown in the purple box. The compounds contained in the purple boxes were significantly more abundant in RX-01 and could be used for identifying the characteristic VOCs of different irradiation intensities for octanol, amyl hexanoate, bornyl acetate, and (E)-2-hexenal, whereas the VOCs in the unboxed area showed a small difference in the content of VOCs among the four controls, thus making it difficult for them to be used as a VOCs to differentiate among the four treatments.

### 2.4. Chemometric Analysis

Chemometrics is a discipline that establishes a relationship between the measured values of chemical systems and the state of the system through statistical or mathematical methods. Applied mathematics, statistics, and computer science can obtain the composition, structure, and other relevant information of the material system to the maximum extent through the processing and analysis of measurement data and they are widely used in traditional Chinese medicine, food, medicine, and other fields.

#### 2.4.1. Principal Component Analysis (PCA)

**Figure 6:**
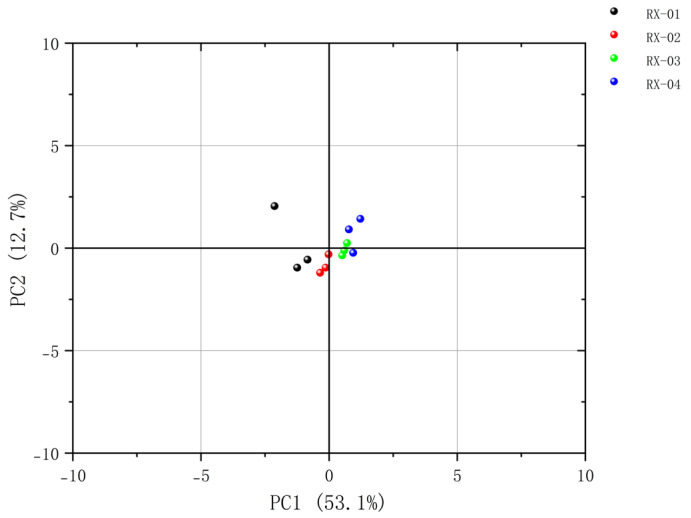
Plot of the PCA scores of the VOCs in the four groups of olibanum.

Principal component analysis (PCA) is one of the oldest multivariate techniques in statistics. When applied to regression analysis, a large number of potentially related variables can be summarized into a representative set of non-correlated variables, which can be used as an important tool for dimensionality reduction or large-scale data visualization [19]. This study used Originpro 2023b software to conduct the principal component analysis of the volatile organic compounds in olibanum after ^60^Co irradiation with different doses. The principal component scores were arranged from high to low according to the contribution rate and the scores of the first two principal components were visually analyzed, the results of which are shown in Figure 6. As the ^60^Co irradiation increased, the distance between each sample became larger and larger, thus indicating that the differences between the VOCs in the samples became more significant. The distance between Samples 1 and 2 was the smallest. This indicated that the appropriate irradiation dose was found in Sample 2 because it had the smallest impact on the substances in olibanum and, thus, its quality.

#### 2.4.2. Cluster Analysis (CA)

**Figure 7:**
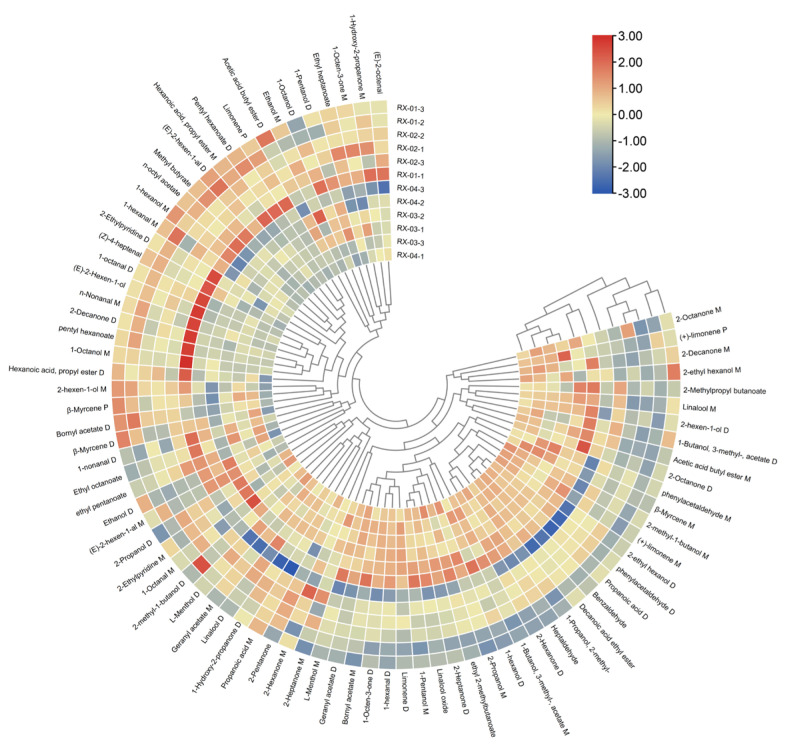
Cluster heat map of the VOCs in the four groups of olibanum.

Cluster analysis (CA) is the grouping of data objects based on the information found in the data describing the objects and their relationships [20]. In order to further build the identification model, this study used heat maps to visualize the data between samples and imported 81 volatile component peaks in olibanum after irradiation at 0 kGy, 1.5 kGy, 3.0 kGy, and 6.0 kGy ^60^Co doses into TBtools. The cluster analysis is depicted in Figure 7. It can be seen that RX-01 and RX-02 were in the same category and that RX-03 and RX-04 were also in the same category.

#### 2.4.3. Partial Least-Squares Discriminant Analysis (PLS-DA)

**Figure 8:**
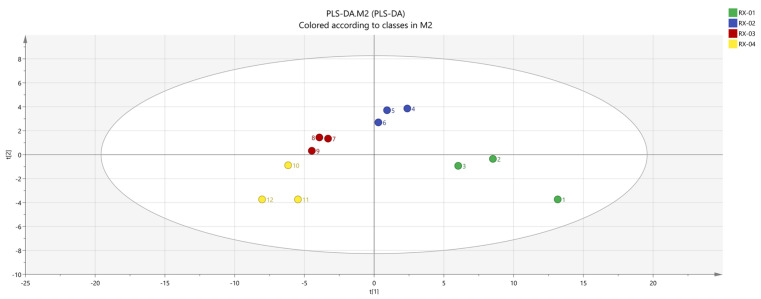
PLS−DA analysis of the VOCs in the four groups of olibanum.

SIMCA was used to conduct a partial least squares discriminant analysis (PLS-DA) [21] of each sample with a supervised pattern recognition method to observe the differences in olibanum after ^60^Co irradiation at 0 kGy, 1.5 kGy, 3 kGy, and 6 kGy doses. The results are shown in Figure 8. It can be seen from the figure that the main areas of olibanum irradiated with different doses do not intersect with each other and are clearly distinguished. RX-01 and RX-02 are both on the right side of the coordinate axis and the distance is farther than the other samples. This indicates that the difference in the VOCs is small, which is consistent with the PCA results. Additionally, according to the processed data, R^2^X = 0.878, R^2^Y = 0.992, and Q^2^ = 0.894, when R^2^ and Q^2^ are greater than 0.5, this indicates that the model has relatively accurate generalization and predictive abilities.

**Figure 9:**
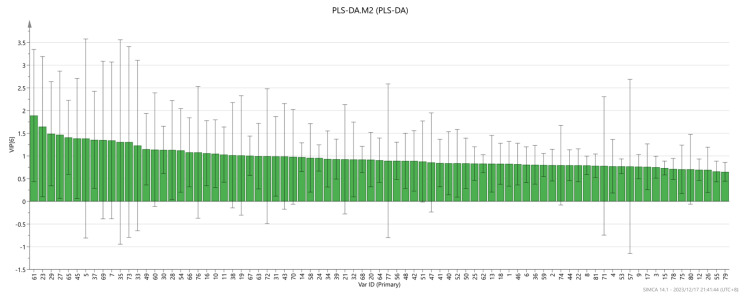
VIP values of the characteristic variables.

In addition, a variable projection importance map was also drawn. The results are shown in Figure 9. It is generally believed that the larger the VIP value, the more important it is. When the VIP value is greater than one, the variable is a more important value, and when the VIP value is less than 0.5, the variable is an unimportant value. It can be seen from the figure that (+)-limonene P, 2-Ethylpyridine M, β-Myrcen P, 2-Decanone M, Ethyl octanoate, β-Myrcene M, 2-Pentanone, (E)-2-hexen-1-al M, Ethyl heptanoate, 2-ethyl hexanol M, 2-Propanol D, 2-Hexanone M, Ethanol D, β-Myrcene D, 2-Methylpropyl butanoate, 1-Octen-3-one M, 1-Hydroxy-2-propanone D, Limonene D, 2-Propanol M, 2-Octanone D, Bornyl acetate D, (Z)-4-heptenal, L-Menthol D, phenylacetaldehyde M, ethyl pentanoate, 1-Propanol, and the VIP value of these substances are all greater than one. In addition, there are also important components that affect the differences between the groups of olibanum after different doses of irradiation.

**Figure 10:**
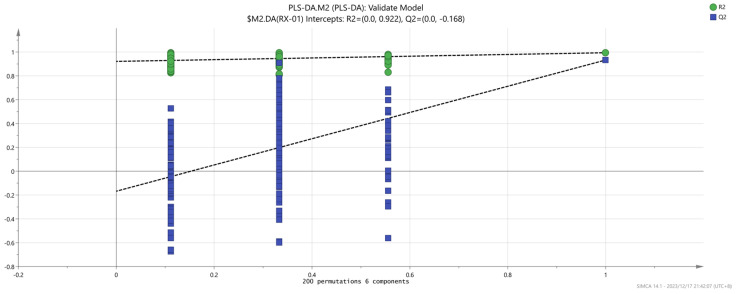
Permutation test results of the VOCs in the four groups of olibanum.

At the same time, in order to judge whether the model is overfitted, we conducted 200 cross-validations to examine both the R^2^ and Q^2^ values. The results found no signs of overfitting (R^2^ = 0.922, Q^2^ = −0.168, as shown in Figure 10).

## 3. Discussion

This study used GC-IMS to detect a total of 81 types of VOCs, including alcohols, esters, aldehydes, ketones, terpenes, olefins, pyridines, and acids. First, the substance contents in the different groups of olibanum were compared and then PCA, CA, and PLS-DA were applied to analyze the four groups of olibanum. It was concluded that the volatile organic compounds of the four groups of olibanum were similar in composition but significantly different in content. In the four groups of olibanum, the contents of olibanum volatile oil esters, ketones, and alcohols, followed by aldehydes, were higher. Additionally, as the irradiation intensity increased, the contents of each volatile organic compound also changed and their contents differed in each sample group. According to previous literature reports, active ingredients such as terpenes and esters in olibanum volatile oil have analgesic, sedative, and antibacterial effects [22]. Most of these main ingredients did not significantly change due to the influence of irradiation, thereby indicating that ^60^Co irradiation sterilization would be predicted to have little effect on the efficacy of the VOCs in olibanum.

By analyzing the PCA results, it was found that PC1 and PC2 were 53.1% and 12.7%, respectively, and the cumulative contribution rate was 65.8%. The PCA results confirmed that there was a significant difference between the control group and the other three groups and the samples in each group were relatively different. After clustering, the repeatability was good in each group, the data similarity was high, and the results were relatively accurate. The results of PLS-DA were clearly separated among the four groups, thereby indicating that the differences between the groups are large. According to the results of the clustering heat map, it could be seen that RX-01 and RX-02 were closer and could be grouped into one category. 

The important materials in the powder of traditional Chinese medicine have the same origins as medicine and food. Mycotoxins and harmful toxins may be present in raw medicinal materials and they may be directly transferred to preparations or foods; therefore, they must be sterilized before use [23]. Due to the heat-labile nature of olibanum itself, compared with the traditional heat sterilization method of traditional Chinese medicine, radiation sterilization reduces the difficulty of sterilization by avoiding the increase in viscosity of olibanum when heated. Compared with traditional heat sterilization, this method is more convenient, faster, and less expensive. The temperature changes during the sterilization process are small and it is suitable for sterilizing the volatile components of heat-sensitive traditional Chinese medicine. Compared with ultraviolet sterilization, radiation sterilization is more thorough and it is not limited to surface sterilization. Radiation sterilization can control the growth of microorganisms or kill microorganisms in a specific manner. Radiation sterilization is a highly safe process and it also produces an excellent sterilization effect. It can also retain the main active ingredients in olibanum while sterilizing. However, if the irradiation dose is too high, this may cause changes in some of the active ingredients in the olibanum.

GC-IMS is often used in the food industry [24], traditional Chinese medicine [25,26,27,28,29,30,31], the agricultural industry [32], and other fields [33]. In this study, VOCs such as esters, ketones, and alcohols were effectively identified by GC-IMS. This facilitated the qualitative and quantitative analyses. The “Technical Guidelines for Radiation Sterilization of Traditional Chinese Medicines” issued by the State Food and Drug Administration in 2017 stipulate that the maximum overall average irradiation dose of traditional Chinese medicines should in principle not exceed 10 kGy; therefore, on the premise of ensuring the full sterilization of olibanum, where the aim is to maintain the original properties of the olibanum medicinal materials, the changes in VOCs after irradiation were minimized, we selected a radiation dose of 1.5 kGy for the irradiation sterilization of olibanum. This study used GC-IMS to determine the content of the four groups of olibanum after irradiation and established a more efficient and convenient operation method, which provided the feasibility of applying ^60^Co irradiation sterilization to olibanum sterilization. Furthermore, we selected a radiation dose of 1.5 kGy as optimal for the irradiation sterilization of olibanum. In future research, we will further explore whether irradiation affects other physical and chemical properties or pharmacological activities.

## 4. Materials and Methods

### 4.1. Materials

Olibanum was provided by Chongqing Healn Drug Sales Co., Ltd., Chongqing, China. A voucher specimen (HNATCM2023-006) was stored in the sample room of the Science and Technology Innovation Center of the Hunan University of Chinese Medicine.

### 4.2. ^60^Co-γ Irradiation

Firstly, the olibanum was crushed into a powder at a low temperature (approximately 4 °C) and then divided into four equal parts for ^60^Co irradiation. The dose rates were 0, 1.5, 3.0, and 6.0 kGy/min. The ^60^Co γ radiation source was located at the Hunan Radiological Technology Application Research Center (Changsha, China).

### 4.3. Analysis by GC–IMS

#### 4.3.1. Sample Preparation

A 20 mL headspace vial was filled with the powder samples of each sample and incubated for 15 min at 80 °C.

#### 4.3.2. Headspace Conditional

The static headspace autosampler unit (CTC-PAL 3), which was manufactured by the CTC Analytics AG in Zwinger, Switzerland, allowed for the injection of 500 L of a headspace non-shunt injection, as well as a rotational speed of 500 revolutions per minute (rpm)\for 20 min. The injection needle temperature was 85 °C.

#### 4.3.3. GC Conditional

MXT-WAX (15 m × 0.53 mm × 1.0 m, Restek Inc., Edmond, OK, USA) was used for chromatography. The column temperature was 60 °C. High-purity N_2_ (purity ≥ 99.999%) was used as the carrier gas. Moreover, the initial flow rate was held for 2 min at 2.00 mL/min. This was increased linearly to 10.00 mL/min within 8 min, then to 100.00 mL/min within 10 min, and was then held for 10 min.

The chromatography runtime was 30 min and the injection temperature was 80 °C; the run time was 30 min and the inlet temperature was 80 °C.

#### 4.3.4. IMS Conditional

The instrument used in this study was the FlavorSpec^®^ Gas Phase Ion Mobility Spectrometer from GAS (Dortmund, Germany); the ionization source was tritium (3H); the drift tube length was 53 mm; the electric field intensity was 500 V/cm; the drift tube temperature was 45 °C; the drift gas was a high-purity N_2_ (99.999%); the flow rate was 150 mL/min; and a positive ion mode was used.

### 4.4. Statistical Analysis

A qualitative and quantitative analysis of the spectrum and data was conducted using the analysis software Vocal, which was paired with the instrument. In order to analyze the substances qualitatively, the application software included the NIST and IMS databases. Using the Reporter plugin, we directly compared the spectrum differences between the samples (three-dimensional spectra, two-dimensional top views, and the difference spectra). Using the Gallery plot plug-in, we compared the fingerprints of different samples to intuitively and quantitatively compare the VOCs. Originpro 2023b software was used for principal component analysis (PCA), TBtools v2.026 was used for cluster analysis, and SIMCA 14.1 was used to conduct a partial least squares discriminant analysis (PLS-DA).

## 5. Conclusions

In this study, olibanum that was irradiated with different doses of ^60^Co was analyzed using GC-IMS. A total of 81 VOCs were detected, including 20 alcohols, 20 esters, 14 aldehydes, 13 ketones, 7 terpenes, 3 alkenes, 2 pyridines, and 2 acids. By establishing fingerprints through the characteristic components fitted by the Gallery plot plug-in software, the olibanum was found to contain the same VOCs after irradiation at different doses but their contents differed significantly. As the irradiation dose increased, the phenylacetaldehyde, 1-Octen-3-one, and limonene substances gradually increased, while the content of Pentyl hexanoate, 1-Octanone, (E)-2-hexen-1-al, and other substances decreased. In addition, statistical analyses such as principal component analysis (PCA), cluster analysis (CA), and partial least squares discriminant analysis (PLS-DA) also confirmed that the higher the dose of ^60^Co irradiation, the greater the changes in olibanum.

From this research, the results demonstrated that 1.5 kGy is a relatively good sterilization dose. This study provides a fast and efficient method for the analysis and evaluation of VOCs in olibanum and it also provides a good reference for irradiation technology development and application to functional foods, making it both significant from a research perspective and useful from an application perspective.

GC-IMS coupled with chemometrics offers several advantages:

- Rapid detection and analysis—this method saves time and money by allowing samples to be detected and analyzed quickly without requiring complex sample processing;
- High sensitivity and accuracy—GC-IMS is highly sensitive and can detect trace components and it can be used to analyze samples qualitatively and quantitatively when combined with chemometric methods;
- Visualization and fingerprint recognition—using this technique, fingerprint spectra can be quickly generated and samples can be compared and identified more easily;
- Multi-sample classification and clustering analysis—based on the differences of VOCs, this method can classify and cluster samples with different processing methods, thereby supporting sample quality control and optimization.

## Figures and Tables

**Table 1:** Comparison of the commonly used sterilization methods.

Sterilization Methods	Advantages	Disadvantages
Dry heat sterilization	(1) It is suitable for items that are resistant to high temperatures.	(1) Compared with moist heat sterilization, the sterilization effect is poor.
Saturated steam sterilization	(1) It has a good sterilization effect and is suitable for items that should not be changed or damaged when exposed to high temperatures and humidity.	(1) Chinese medicine powder is likely to absorb moisture and the sterilization, which is conducted using saturated steam, is more likely to cause it to agglomerate, which may increase the time required for the drying process or cause secondary pollution.
Drug sterilization	(1) It can reduce the number of microorganisms and provide a certain level of sterility. (2) It is suitable for surface sterilization and environmental sterilization.	(1) It is only effective on microbial reproductive bodies and cannot kill spores. (2) There is a risk of drug residues. (3) It is mostly used for sterilizing pieces and medicinal materials and there is almost no sterilization effect for powders.
Ultraviolet sterilization	(1) Ultraviolet sterilization is the most suitable for surface sterilization and environmental sterilization.	(1) Other than surface sterilization and environmental sterilization, other sterilization is not often used.

**Table 2:** Results of the component analysis of VOCs in olibanum.

Count	Compound	CAS#	Molecular Formula	MW	RI	Rt/s	Dt/ms
1	pentyl hexanoate	C540078	C_11_H_22_O_2_	186.3	1510.4	979.146	2.17211
2	Pentyl hexanoate D	C540078	C_11_H_22_O_2_	186.3	1528.7	1017.409	1.53722
3	n-octyl acetate	C112141	C_10_H_20_O_2_	172.3	1483.9	926.536	2.16943
4	2-Decanone D	C693549	C_10_H_20_O	156.3	1499.5	957.145	2.00276
5	2-Decanone M	C693549	C_10_H_20_O	156.3	1503.9	965.997	1.46578
6	Benzaldehyde	C100527	C_7_H_6_O	106.1	1519.3	997.478	1.14899
7	Ethyl octanoate	C106321	C_10_H_20_O_2_	172.3	1462.6	886.258	1.47563
8	Linalool oxide	C1365191	C_10_H_18_O_2_	170.3	1452.1	867.082	1.2651
9	Bornyl acetate M	C76493	C_12_H_20_O_2_	196.3	1577.5	1126.339	1.23104
10	Bornyl acetate D	C76493	C_12_H_20_O_2_	196.3	1583.2	1139.67	2.17616
11	(Z)-4-heptenal	C6728310	C_7_H_12_O	112.2	1245.2	530.841	1.60822
12	2-methyl-1-butanol M	C137326	C_5_H_12_O	88.1	1208.3	470.58	1.23368
13	Geranyl acetate M	C105873	C_12_H_20_O_2_	196.3	1724.9	1531.62	1.22697
14	Geranyl acetate D	C105873	C_12_H_20_O_2_	196.3	1728	1541.562	1.89759
15	L-Menthol M	C2216515	C_10_H_20_O	156.3	1681.3	1398.396	1.22874
16	L-Menthol D	C2216515	C_10_H_20_O	156.3	1654.6	1322.836	1.88697
17	Decanoic acid ethyl ester	C110383	C_12_H_24_O_2_	200.3	1664.6	1350.674	1.62156
18	phenylacetaldehyde D	C122781	C_8_H_8_O	120.2	1603.7	1189.611	1.52955
19	phenylacetaldehyde M	C122781	C_8_H_8_O	120.2	1607.4	1198.733	1.25911
20	1-Octanol M	C111875	C_8_H_18_O	130.2	1591.8	1160.4	1.46872
21	1-Octanol D	C111875	C_8_H_18_O	130.2	1584.7	1143.363	1.88349
22	2-Ethylpyridine D	C100710	C_7_H_9_N	107.2	1280.7	596.272	1.46193
23	2-Ethylpyridine M	C100710	C_7_H_9_N	107.2	1282.9	600.603	1.09944
24	Heptaldehyde	C111717	C_7_H_14_O	114.2	1180.5	429.092	1.33291
25	1-hexanal D	C66251	C_6_H_12_O	100.2	1098.4	324.74	1.56213
26	1-hexanal M	C66251	C_6_H_12_O	100.2	1089.3	315.53	1.25353
27	β-Myrcene M	C123353	C_10_H_16_	136.2	1166.8	409.562	1.2263
28	β-Myrcene D	C123353	C_10_H_16_	136.2	1166.4	409.008	1.65043
29	β-Myrcene P	C123353	C_10_H_16_	136.2	1167.2	410.117	1.72986
30	2-Methylpropyl butanoate	C539902	C_8_H_16_O_2_	144.2	1161.5	402.204	1.33163
31	1-Butanol, 3-methyl-, acetate M	C123922	C_7_H_14_O_2_	130.2	1141.9	376.315	1.3017
32	1-Butanol, 3-methyl-, acetate D	C123922	C_7_H_14_O_2_	130.2	1141.9	376.315	1.74601
33	2-Pentanone	C107879	C_5_H_10_O	86.1	1016.5	251.56	1.12223
34	Methyl butyrate	C623427	C_5_H_10_O_2_	102.1	981.4	227.629	1.13716
35	(E)-2-hexen-1-al M	C6728263	C_6_H_10_O	98.1	1209.4	472.227	1.18215
36	(E)-2-hexen-1-al D	C6728263	C_6_H_10_O	98.1	1207	468.546	1.52541
37	Ethyl heptanoate	C106309	C_9_H_18_O_2_	158.2	1306.3	639.738	1.91244
38	ethyl pentanoate	C539822	C_7_H_14_O_2_	130.2	1106.1	333.321	1.69079
39	ethyl 2-methylbutanoate	C7452791	C_7_H_14_O_2_	130.2	1040	270.691	1.66629
40	Propanoic acid M	C79094	C_3_H_6_O_2_	74.1	1567.8	1103.773	1.10089
41	Propanoic acid D	C79094	C_3_H_6_O_2_	74.1	1569.6	1108.025	1.26778
42	Linalool D	C78706	C_10_H_18_O	154.3	1580.5	1133.374	1.76497
43	Linalool M	C78706	C_10_H_18_O	154.3	1573.7	1117.326	1.20039
44	2-ethyl hexanol D	C104767	C_8_H_18_O	130.2	1503.4	965.009	1.79735
45	2-ethyl hexanol M	C104767	C_8_H_18_O	130.2	1503.4	965.009	1.42224
46	1-hexanol M	C111273	C_6_H_14_O	102.2	1383.8	752.013	1.32506
47	1-hexanol D	C111273	C_6_H_14_O	102.2	1383.1	750.874	1.63894
48	1-Octen-3-one D	C4312996	C_8_H_14_O	126.2	1350.8	701.962	1.67677
49	1-Octen-3-one M	C4312996	C_8_H_14_O	126.2	1348.3	698.397	1.26328
50	n-Nonanal M	C124196	C_9_H_18_O	142.2	1410.1	794.363	1.47424
51	1-nonanal D	C124196	C_9_H_18_O	142.2	1406.9	789.17	1.95326
52	2-hexen-1-ol M	C2305217	C_6_H_12_O	100.2	1368.1	727.768	1.1783
53	2-hexen-1-ol D	C2305217	C_6_H_12_O	100.2	1362.2	718.886	1.51225
54	1-Hydroxy-2-propanone D	C116096	C_3_H_6_O_2_	74.1	1333	676.427	1.23573
55	1-Hydroxy-2-propanone M	C116096	C_3_H_6_O_2_	74.1	1341.3	688.171	1.03734
56	1-Pentanol M	C71410	C_5_H_12_O	88.1	1290.6	615.901	1.2604
57	1-Pentanol D	C71410	C_5_H_12_O	88.1	1288.1	611.034	1.51755
58	2-methyl-1-butanol D	C137326	C_5_H_12_O	88.1	1215.9	482.362	1.47147
59	(+)-limonene M	C138863	C_10_H_16_	136.2	1207.4	469.193	1.21097
60	Limonene D	C138863	C_10_H_16_	136.2	1215.9	482.395	1.29868
61	(+)-limonene P	C138863	C_10_H_16_	136.2	1224.4	496.001	1.65214
62	Limonene P	C138863	C_10_H_16_	136.2	1224.9	496.756	1.71661
63	2-Heptanone M	C110430	C_7_H_14_O	114.2	1192.5	446.871	1.25839
64	2-Heptanone D	C110430	C_7_H_14_O	114.2	1192	446.115	1.63832
65	2-Propanol D	C67630	C_3_H_8_O	60.1	950.7	210.887	1.20859
66	2-Propanol M	C67630	C_3_H_8_O	60.1	947.7	209.365	1.09106
67	1-Propanol, 2-methyl-	C78831	C_4_H_10_O	74.1	1057.1	285.441	1.38133
68	2-Hexanone D	C591786	C_6_H_12_O	100.2	1088.5	314.769	1.48915
69	2-Hexanone M	C591786	C_6_H_12_O	100.2	1093.5	319.657	1.1786
70	Acetic acid butyl ester M	C123864	C_6_H_12_O_2_	116.2	1122.9	352.895	1.22652
71	Acetic acid butyl ester D	C123864	C_6_H_12_O_2_	116.2	1118	347.029	1.60157
72	Ethanol M	C64175	C_2_H_6_O	46.1	951.8	211.489	1.04662
73	Ethanol D	C64175	C_2_H_6_O	46.1	951.5	211.35	1.11704
74	(E)-2-octenal	C2548870	C_8_H_14_O	126.2	1423.5	816.94	1.32526
75	(E)-2-Hexen-1-ol	C928950	C_6_H_12_O	100.2	1443.3	851.454	1.51189
76	2-Octanone D	C111137	C_8_H_16_O	128.2	1271.1	577.882	1.77106
77	2-Octanone M	C111137	C_8_H_16_O	128.2	1272.4	580.411	1.33372
78	1-octanal D	C124130	C_8_H_16_O	128.2	1322.2	661.348	1.83779
79	1-Octanal M	C124130	C_8_H_16_O	128.2	1317.2	654.506	1.39805
80	Hexanoic acid, propyl ester D	C626777	C_9_H_18_O_2_	158.2	1305.2	638.326	1.86479
81	Hexanoic acid, propyl ester M	C626777	C_9_H_18_O_2_	158.2	1303.5	636.014	1.39506

**Table 3:** The average area of VOCs in olibanum.

Count	Compound	CAS#	Molecular Formula	RX-1	RX-2	RX-3	RX-4
1	pentyl hexanoate	C540078	C_11_H_22_O_2_	6166.21	4832.44	4562.9	4525.01
2	Pentyl hexanoate D	C540078	C_11_H_22_O_2_	19689.2	18019	16901.5	16726.6
3	n-octyl acetate	C112141	C_10_H_20_O_2_	11749.2	11611.5	11459.6	11371.2
4	2-Decanone D	C693549	C_10_H_20_O	10632	10281.3	10192.4	10197.2
5	2-Decanone M	C693549	C_10_H_20_O	374.511	338.806	404.039	414.96
6	Benzaldehyde	C100527	C_7_H_6_O	709.125	1025.71	1200.96	1199.4
7	Ethyl octanoate	C106321	C_10_H_20_O_2_	1172.34	1172.34	1202.74	1151.07
8	Linalool oxide	C1365191	C_10_H_18_O_2_	2204.96	2396.34	2593.39	2700.51
9	Bornyl acetate M	C76493	C_12_H_20_O_2_	9335.95	9498.94	9641.87	9695.36
10	Bornyl acetate D	C76493	C_12_H_20_O_2_	979.352	959.329	936.968	876.834
11	(Z)-4-heptenal	C6728310	C_7_H_12_O	15085.3	14809.7	14686.9	14807.7
12	2-methyl-1-butanol M	C137326	C_5_H_12_O	1678.08	1793.71	1870.12	1910.35
13	Geranyl acetate M	C105873	C_12_H_20_O_2_	13495.2	14007.2	14205.4	14030.5
14	Geranyl acetate D	C105873	C_12_H_20_O_2_	611.904	663.192	782.069	780.187
15	L-Menthol M	C2216515	C_10_H_20_O	18963.4	19364.7	19792.6	19953.5
16	L-Menthol D	C2216515	C_10_H_20_O	718.083	845.315	804.558	852.996
17	Decanoic acid ethyl ester	C110383	C_12_H_24_O_2_	801.752	1043.14	1185.94	1178.22
18	phenylacetaldehyde D	C122781	C_8_H_8_O	961.065	1197.63	1240.98	1258.83
19	phenylacetaldehyde M	C122781	C_8_H_8_O	257.619	285.83	319.445	301.018
20	1-Octanol M	C111875	C_8_H_18_O	2441.74	2150.02	2076.51	2156.68
21	1-Octanol D	C111875	C_8_H_18_O	2381.87	2433.27	2397.48	2369.82
22	2-Ethylpyridine D	C100710	C_7_H_9_N	833.436	796.274	777.981	786.681
23	2-Ethylpyridine M	C100710	C_7_H_9_N	438.986	463.811	436.835	480.888
24	Heptaldehyde	C111717	C_7_H_14_O	2188.73	2371.51	2382.34	2460.87
25	1-hexanal D	C66251	C_6_H_12_O	183.503	206.681	227.375	230.236
26	1-hexanal M	C66251	C_6_H_12_O	177.585	170.648	167.713	167.05
27	β-Myrcene M	C123353	C_10_H_16_	1326.28	1350.92	1420.17	1377.39
28	β-Myrcene D	C123353	C_10_H_16_	749.866	714.263	730.025	706.984
29	β-Myrcene P	C123353	C_10_H_16_	566.134	545.741	558.405	521.838
30	2-Methylpropyl butanoate	C539902	C_8_H_16_O_2_	144.023	151.716	150.365	166.674
31	1-Butanol, 3-methyl-, acetate M	C123922	C_7_H_14_O_2_	1137.22	1164.71	1204.89	1194.04
32	1-Butanol, 3-methyl-, acetate D	C123922	C_7_H_14_O_2_	106.119	103.849	106.773	114.743
33	2-Pentanone	C107879	C_5_H_10_O	8824.47	9050.13	8928.28	8841.22
34	Methyl butyrate	C623427	C_5_H_10_O_2_	1577.17	1539.72	1484.63	1494.26
35	(E)-2-hexen-1-al M	C6728263	C_6_H_10_O	293.772	270.11	277.362	293.657
36	(E)-2-hexen-1-al D	C6728263	C_6_H_10_O	528.104	480.33	443.145	413.408
37	Ethyl heptanoate	C106309	C_9_H_18_O_2_	4723.98	4741.77	4687.61	4570.13
38	ethyl pentanoate	C539822	C_7_H_14_O_2_	4341.31	4355.82	4340.36	4316.53
39	ethyl 2-methylbutanoate	C7452791	C_7_H_14_O_2_	584.269	623.721	631.426	663.152
40	Propanoic acid M	C79094	C_3_H_6_O_2_	782.116	829.134	826.948	805.403
41	Propanoic acid D	C79094	C_3_H_6_O_2_	459.302	579.502	607.948	600.481
42	Linalool D	C78706	C_10_H_18_O	651.32	701.026	696.31	699.998
43	Linalool M	C78706	C_10_H_18_O	882.766	871.122	960.994	981.299
44	2-ethyl hexanol D	C104767	C_8_H_18_O	439.375	557.98	580.149	615.102
45	2-ethyl hexanol M	C104767	C_8_H_18_O	170.445	143.923	174.288	165.093
46	1-hexanol M	C111273	C_6_H_14_O	1158.82	1136.34	1112.27	1087.17
47	1-hexanol D	C111273	C_6_H_14_O	744.558	768.731	790.571	783.4
48	1-Octen-3-one D	C4312996	C_8_H_14_O	1028.76	1088.56	1143.76	1136.71
49	1-Octen-3-one M	C4312996	C_8_H_14_O	761.036	764.74	757.642	731.007
50	n-Nonanal M	C124196	C_9_H_18_O	1411.83	1336.42	1332.85	1318.52
51	1-nonanal D	C124196	C_9_H_18_O	824.871	817.941	837.099	825.528
52	2-hexen-1-ol M	C2305217	C_6_H_12_O	6366.4	6305.74	6260.07	6209.13
53	2-hexen-1-ol D	C2305217	C_6_H_12_O	646.994	678.048	718.526	725.791
54	1-Hydroxy-2-propanone D	C116096	C_3_H_6_O_2_	1580.08	1754.46	1758.74	1687.98
55	1-Hydroxy-2-propanone M	C116096	C_3_H_6_O_2_	398.082	390.351	377.894	372.725
56	1-Pentanol M	C71410	C_5_H_12_O	1692.33	1745.98	1789.17	1846.58
57	1-Pentanol D	C71410	C_5_H_12_O	734.164	731.392	737.944	727.874
58	2-methyl-1-butanol D	C137326	C_5_H_12_O	184.259	178.81	177.609	190.024
59	(+)-limonene M	C138863	C_10_H_16_	3811.6	4116.81	4352.17	4622.78
60	Limonene D	C138863	C_10_H_16_	275.468	287.89	319.194	310.758
61	(+)-limonene P	C138863	C_10_H_16_	3639.86	3594.6	3702.99	3648.12
62	Limonene P	C138863	C_10_H_16_	1813.81	1742.59	1648.43	1641.64
63	2-Heptanone M	C110430	C_7_H_14_O	404.007	426.06	431.479	419.199
64	2-Heptanone D	C110430	C_7_H_14_O	114.195	134.984	146.059	166.078
65	2-Propanol D	C67630	C_3_H_8_O	682.751	768.506	722.871	774.708
66	2-Propanol M	C67630	C_3_H_8_O	755.315	841.222	850.021	937.544
67	1-Propanol, 2-methyl-	C78831	C_4_H_10_O	3808.37	3966.91	4025.48	3998.99
68	2-Hexanone D	C591786	C_6_H_12_O	4367.6	4836.69	4900.91	5077.25
69	2-Hexanone M	C591786	C_6_H_12_O	484.799	517.764	503.058	502.545
70	Acetic acid butyl ester M	C123864	C_6_H_12_O_2_	6989.78	6988.76	7063.67	7144.74
71	Acetic acid butyl ester D	C123864	C_6_H_12_O_2_	1095.62	1094.13	1097.9	1082.23
72	Ethanol M	C64175	C_2_H_6_O	907.333	919.473	905.521	914.475
73	Ethanol D	C64175	C_2_H_6_O	5851.25	5569.31	5786.19	5901.12
74	(E)-2-octenal	C2548870	C_8_H_14_O	1812.36	1813.37	1788.66	1772.13
75	(E)-2-Hexen-1-ol	C928950	C_6_H_12_O	1302.66	1209.32	1163.19	1157.15
76	2-Octanone D	C111137	C_8_H_16_O	1277.29	1266.13	1303.78	1341.46
77	2-Octanone M	C111137	C_8_H_16_O	718.538	722.205	744.743	739.138
78	1-octanal D	C124130	C_8_H_16_O	1494.51	1403.39	1321.47	1290.42
79	1-Octanal M	C124130	C_8_H_16_O	204.751	209.574	214.15	219.14
80	Hexanoic acid, propyl ester D	C626777	C_9_H_18_O_2_	322.2	311.512	306.253	298.796
81	Hexanoic acid, propyl ester M	C626777	C_9_H_18_O_2_	454.561	409.236	391.367	363.406

## Data Availability

Data are contained within the article.
